# 

**Published:** 1913-05-03

**Authors:** 


### Obituary.

Dr. Sydney William Cheetham, of Forest Gate, died suddenly on April 27 at the house of one of his patients, at the comparatively early age of forty-five. He represented the medical profession on the West Ham Insurance Committee, and had practised in the locality for over twenty years.

Dr. Alexander Mitchell, surgeon to the C Division of the Metropolitan Police, has died at Shillingford, aged sixty-four. Educated at Glasgow and Edinburgh, he graduated at the former in 1873, and M.D. three years later. His earlier appointments included those of medical officer of health for Great Yarmouth, and for Canisbay, Caithness, house surgeon at the Glasgow Lock Hospital, and honorary physician to the Gorleston Hospital.

### Gifts and Bequests.

The Chelsea Hospital for Women has received £100 in £10 notes from " Z.B.B." towards the rebuilding fund.

The Drapers' Company have sent 25 guineas to the Homes for Little Boys, Farningham and Swanley, Kent.

The Fishmongers' Company have given £26 5s. and the Armourers' and Brasiers' Company a further £10 10s. to the Royal Female Orphan Asylum.

Prince Alexander of Teck has received from Sir Charles W. Morrison-Bell, Bart., £1,000 for the purchase of radium for the Cancer Charity of the Middlesex Hospital.

### Appointments.

Mr. B. Whitchurch Howell, M.B., B.S. (Lond.), M.R.C.S., L.R.C.P. (Lond.), has been appointed house surgeon at the Royal Free Hospital, Gray's Inn Road.

Dr. Mary Brown, of York, has been appointed resident medical officer by the Keighley and Bingley Joint Hospital Board at the infectious diseases hospital at Morton banks.

The Council of the University of Sheffield has appointed Mr. F. G. Mordaunt, L.D.S., to the post of Lecturer in Dental Surgery and Pathology, in succession to the late Mr. Frank Harrison.

Dr. Hayward Willett has been appointed honorary surgeon to the Liverpool Hospital for Women, at which he has been assistant surgeon for ten years. Dr. Willett graduated M.B. with honours at Liverpool University, and later took the M.D. degree, since devoting himself chiefly to obstetrics and gynæcology.

Dr. J. Coote Hibbert, medical officer of health, Warrington, has been appointed medical officer of health, Blackburn, at a salary of £750 per annum. Dr. Hibbert, who is an M.D. of London University, was for six years medical officer at Warrington, and for three years previously had been deputy medical officer at Newcastle. He has paid a visit to India to study the plague and kindred branches of preventive medicine.

